# Vulnerability to alcohol consumption, spiritual transcendence and psychosocial well-being: test of a theory**^1^**

**DOI:** 10.1590/1518-8345.0688.2702

**Published:** 2016-06-07

**Authors:** Luz Patricia Díaz Heredia, Alba Idaly Muñoz Sanchez

**Affiliations:** 2 Associate Professor, Facultad de Enfermería, Universidad Nacional de Colombia , Bogotá, DC, Colombia.; 3 Full Professor, Facultad de Enfermería, Universidad Nacioal de Colombia, Bogotá, DC, Colombia.

**Keywords:** Nursing Theory, Alcohol Drinking, Young Adult

## Abstract

**Objective:**

to demonstrate the relations among vulnerability, self-transcendence and well-being in the young adult population and the effect of each of these variables on the adoption of low-risk consumption conducts.

**Method:**

quantitative and cross-sectional correlation study using structural equations analysis to test the relation among the variables.

**Results:**

an inverse relation was evidenced between vulnerability to alcohol consumption and spiritual transcendence (β-0.123, p 0.025) and a direct positive relation between spiritual transcendence and psychosocial well-being (β 0.482, p 0.000).

**Conclusions:**

the relations among the variables spiritual transcendence, vulnerability to alcohol consumption and psychosocial well-being, based on Reed's Theory, are confirmed in the population group of young college students, concluding that psychosocial well-being can be achieved when spiritual transcendence is enhanced, as the vulnerability to alcohol consumption drops.

## Introduction 

This paper is part of the Doctoral dissertation *Self-transcendence, spirituality and well-being in college students not consuming and moderately consuming alcohol: Contributions to health promotion,* in which the need was raised to determine the relations among the variables spirituality, self-transcendence and well-being and low-risk consumption patterns, evidenced by the alcohol consumption levels of young college students in a particular phase of life called "emerging adulthood", based on Reed's theoretical proposal of self-transcendence^1^. Emerging adulthood is a development phase that includes the period from 18 till 25 years and is characterized by the exploration of one's identity, instability regarding the position in life, self-centeredness and having a large number of possibilities and projects in life^2^, corresponding to young adult college students in this study. In health promotion, nursing has a lot to offer, due to its integrative orientation based on care and social commitment^3^; on the other hand, because nursing has a solid knowledge base that allows it to question aspects of care for human beings' health experience^4^, a perspective that is interested in understanding the development and place of human beings in the world, in combination with the desire to know the topics that strengthen the adoption and maintenance of positive health conducts. As such, well-being, self-transcendence and vulnerability, which are the central concepts in Pamela Reed's theory of Self-transcendence, served to structure the study. The theory of self-transcendence is a midrange theory of nursing, developed to understand the nature of human beings' growth and the relevance of the phenomenon of development and well-being. The purpose of the theory is to gain further understanding on well-being in the adult phase or in the process of reaching adulthood. Self-transcendence is a broad characteristic of maturity, in terms of the broader or better awareness of the environment and a broader perspective of life. The core propositions establish that self-transcendence is related with situations that confront people with the finiteness of existence or at important times of internal change. The theory expresses that the self-conceptual limits are related to well-being. Depending on its nature, the fluctuations that take the form of broader limits influence the well-being positive or negatively in the course of the lifecycle. The relation between vulnerability and self-transcendence is not linear and is not related to low or high levels of vulnerability. There are factors that can affect it, such as personal and contextual factors. Self-transcendence is direct and positively related with well-being^5^.

Spirituality factor is a moderating factor of behavior and a mechanism to prevent alcohol consumption, as well as a predictor and recovery factor of alcohol abuse^5^ ^-^ ^6^. Religion and spirituality play an important role during emerging adulthood when a particular religious orientation is present^6^. 

In the literature, a strong positive relation is presented between spirituality and the health level in college students^6^. The protective effects are mediated by negative beliefs regarding alcohol, social modeling and the reasons to consume alcohol, but cannot always be considered as protective factors^7^. The relations between spirituality and alcohol consumption are not consistently supported, therefore, the recommendation is to continue studies with a view to further clarifications in this population group^8^ ^-^ ^9^.

Concerning the variable self-transcendence, based on the studies considered in the review, a positive relation can be evidenced with emotional, physical, spiritual and social well-being^10^. It is present in alcohol abusers who recovered. Levels of spiritual well-being are higher in college students who do not consume alcohol and it is considered a possible intervention to reduce alcohol consumption^11^.

A negative association exists between the level of alcohol consumption and the well-being of adults, particularly due to the presence of depression and anxiety^12^. The positive association between moderate consumption and well-being in men disappears when sociodemographic variables like age, education etc. are included. Young adult women present a higher level of social and educative well-being than non-consuming adults^12^. 

Young people who do not consume alcohol, practice a religion and have a higher socioeconomic level present higher levels of psychological well-being^13^. In young adults, a positive association was found among spirituality, continued abstinence and the level of psychological well-being^14^. According to the literature, there is no conclusive evidence yet on the relations among the variables. Therefore, it is important to test whether the proposals of the theory of self-transcendence are proven in this group of young people with low-risk alcohol consumption. The objective in this study was to empirically demonstrate the relations proposed in Reed's theory of Self-transcendence among vulnerability, self-transcendence and well-being, based on the data of a young adult population that does not or moderately consumes alcohol. 

## Method

Quantitative, non-experimental, cross-sectional correlation study developed at a national public university with a population of 25,000 students on the campus where the study was developed. A stratified, random, proportional sample was obtained of 499 students who complied with the following inclusion criteria: being a student enrolled at Universidad Nacional de Colombia on the Bogotá Campus and between 18 and 25 years of age, having scored 0 on the AUDIT-C and CAGE screenings tests to be considered a non-consumer and up to 3 for women and 4 for men on the AUDIT-C and 1 on the CAGE for moderate consumers. Students who had been treated for alcohol addiction or had been alcohol abusers were excluded, even if they currently were abstemious. Sampling was based on a finite population and the confidence level was set at α =0.05, with a proportion of non-consumers of 3% based on the pilot test and a proportion of consumers corresponding to 32%. Precision was considered as 3% for non-consumers and 5% for moderate consumers. For the sake of randomization, the list of students enrolled per faculty and gender between 18 and 25 years of age was considered, which for the study period corresponded to 18,971 students. Based on the randomized list produced in Excel, departing from the response rate to the pilot test, it was established that 4,000 students had to be invited. These were invited by e-mail, resulting in 1,010 full answers, to which the inclusion and exclusion criteria were applied, resulting in the final sample of 139 non-consumers and 360 moderate alcohol consumers. The data were collected through the Internet in the first semester of 2011. The following tools were used in the study: the Self Transcendence Scale (STS) designed by Reed^16^ to measure how people expand their personal limits in different ways; the tool consists of 15 items assessed on a four-point Likert scale. The reliability, determined using Cronbach's alpha, originally corresponded to 0.80 for the English version and to 0.77 for a Korean version in 2007. In this study, the Cronbach's alpha coefficient corresponded to 0.85. 

Keyes' Scale of Social Well-being, developed in 1998^17^ ^-^ ^18^, assesses five subscales: social integration, social acceptance, social contribution, social actualization and social coherence; and consists of 33 items on a Likert scale ranging from 1 (strong disagreement) to 6 (strong agreement). The results obtained for internal consistency in the study of the Spanish version ranged between 0.68 and 0.83 and, for this study, the reliability coefficient amounted to 0.74. 

Ryff's Scale of Psychological Well-Being^19^, which consists of six scales and 29 items assessed on a Likert scale between 1 (strong disagreement) and 6 (strong agreement) was used in the Spanish version by Diaz and Blanco^20^, which has demonstrated internal consistency coefficients of 0.84, 0.70 and 0.91 in adolescent populations. In this study, Cronbach's alpha corresponded to 0.89. The Spirituality Questionnaire by Parsian and Dunning^21^ ^-^ ^22^ is focused on the concept of internal identity concept, meaning of life and sense of connection of young people. The subscales of the questionnaire are: Self-awareness, Spiritual beliefs in beliefs, Spiritual practices and Spiritual needs. The construct validity measured by factor analysis revealed four factors that explained 62.7% of the variance, and the internal consistency amounted to 0.94. In this study, the alpha coefficient equaled 0.91.

The authors designed and constructed the vulnerability survey to alcohol consumption for this study based on the literature review. This scale consists of four dimensions: availability of consumption, consumption characteristics and type of consumption. It consists of 10 items, the alpha coefficient obtained in this study for the survey corresponded to 0.60 and the exploratory factor analysis confirmed the four dimensions with an explained variance of 55.33%. All variables measured in the study were processed as discrete ordinal variables for the purpose of statistical analysis of the way their authors recommended them. 

In this study, the ethical principles required for research involving human beings described in the international guidelines for the ethical assessment of epidemiological studies. Ethical approval was obtained from the Research Ethics Committee of the School of Nursing at Universidad Nacional de Colombia. The informed consent tool was completed before answering the tools through an Internet application that, when the acceptance was validated, granted access to the tools. In case of a negative answer, the application issued acknowledgements for the attention paid to the invitation e-mail. The system assigned a number to each participant and stored the information in an Excel matrix. The researchers did not know the participant number. The data analysis was undertaken in the statistical software SPSS 18(r). Structural equations modeling through the generalized least squares method was used, developed in the SPSS software AMOS 6. 

### Data analysis 

The structural equations are a statistical technique from the family of multivariate statistical models that permit estimating the effect and the relations among multiple variables. To develop the analysis, the four phases for the determination of structural equations were followed: identification, estimation of parameters, evaluation of adjustment and re-specification of the model. The identification phase of the model was based on Reed's theoretical proposal. The basic premises to develop the model were assessed, using four variables: vulnerability to consumption, self-transcendence and psychological and social well-being. The estimation phase of the empirical model included the estimation procedure, which was the maximum likelihood procedure (ML). The evaluation of the model included the global estimation of the model through the global adjustment ratios chi-squared, goodness of fit index (GFI) and root mean square error of approximation (RMSEA), besides the determination of the validity and reliability of the proposed model. 

## Results 

Two models were established. The first model consisted of three variables observed: vulnerability to alcohol consumption, self-transcendence and psychological well-being. In this model, the β coefficients of the relations between vulnerability to consumption and self-transcendence (β -0.101, p 0.24) and between self-transcendence and psychological well-being (β 0.185, p 0.00) were statistically significant. To analyze the goodness-of-fit of the proposed model, the chi-squared was established as 0.01 (gl1), p=0.776, (GFI= 1.0), (RMSEA= 0.00), results that indicate proper goodness-of-fit of the data to Reed's theory of Self-transcendence. In this model, however, social well-being and spirituality were not included due to the lack of statistical goodness-of-fit. Therefore, the model was respecified through the construction of two latent variables: spiritual transcendence and psychosocial well-being. 

Thus, the new model with established goodness-of-fit and respecification evidenced that the regression weight β between the variables spiritual transcendence and vulnerability to consumption is significant at 0.05, that is, when the vulnerability to consumption increases, the spiritual transcendence drops. The regression weight β between the variables spiritual transcendence and psychosocial wellbeing is higher than in the first model, with statistical significance at 0.001, as shown in Table 1. The explained variance of psychosocial well-being because of the spiritual transcendence was 23.2%. 

**Table 1:** Standardized regression weights between the variables and the indicators of the latent variables spiritual self-transcendence and psychosocial well-being in the constructed model, Young adult college students, Bogotá, Colombia, 2011.

Research Variables		Standardized regression weight		Standard error	p value
Spiritual transcendence	Vulnerability	-.123	.035	.025*
Psychosocial well-being	Spiritual transcendence	.482	.116	.000†
Spirituality	Spiritual transcendence	.647	.884	.000†
Self-transcendence	Spiritual transcendence	.748	.561	.000†
Psychological well-being	Psychological well-being	.443	.054	.000†
Social well-being	Psychological well-being	.671	.098	.000†

*p< 0.05, †p <0.01

**Figure 1:**
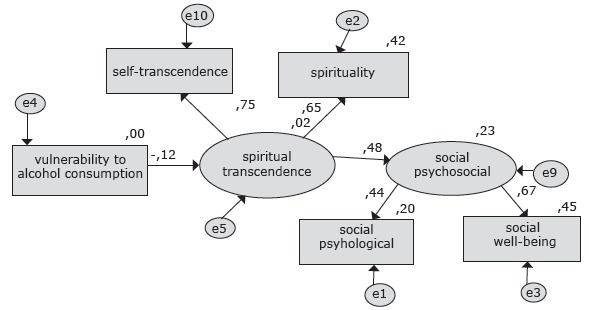
Path Diagram of the expanded model of Reed's theory, including the variables vulnerability to consumption, spiritual transcendence and psychosocial wellbeing in young adult college students, Bogotá, Colombia, 2011

The regression weights λ between the indicators of the latent variable spiritual transcendence were statistically significant with a confidence percentage of 99%, as well as for self-transcendence and spirituality. Concerning the latent variable psychosocial well-being, the regression weights between social well-being and psychological well-being were also significant according to Figure 1. 

The reliability (0.69, 0.50) and extracted variance (0.53, 032) of the latent variables was established and the results reveal that both constructs are constituted appropriately, the spiritual transcendence being more solid. The absolute goodness-of-fit ratios of the model were: Chi-squared 4.262, (gl4) p=0.371, revealing good absolute goodness-of-fit. As for the parsimonious goodness-of-fit ratios, the GFI= 0.997, AGFI 0.987, RMSEA 0.012 and p-value associated PCLOSE 0.813 revealed an appropriate goodness-of-fit of the proposed model to the data collected in the group of young adult college students. 

## Discussion

The obtained results reveal that the relations between the concepts presented in Reed's midrange theory of self-transcendence^15^ ^,^ ^23^ are empirically supported by the data obtained in the sample of non-consuming and moderately consuming emerging adults, in the first as well as in the second model proposed. In the first model, the obtained regression between the variables "vulnerability to consumption" and "self-transcendence" was inverse, signaling that a low vulnerability to consumption is related to higher levels of self-transcendence; according to Reed^16^, with very low or very high levels of vulnerability, the positive relationship between these two variables cannot be evidenced. It would be important to assess, in a group with consumers and higher vulnerability, if the relation is positive and direct as the theorist proposes in situations of great vulnerability. The relation between transcendence and lower alcohol consumption has been reported in a single study with a young adult population in the group of non-consumers of alcohol^24^. This finding manifests that the young people's internal development through different routes, such as art, religion, life experiences and teachings in the family group generate the early development of the ability to expand one's limits, supporting one's decision making and the adoption of conducts that favor health and well-being. 

The relation between the variables self-transcendence and psychological well-being was positive; its direction was the same as in the theory. The same association has been described in different clinical^25^ and community^26^ ^-^ ^27^ spheres, which evidences that self-transcendence sustains and promotes people's mental health and well-being at the different moments in life; by improving the awareness of the environment one lives in and by enhancing the perspective towards existence, strategies are developed to overcome the adversity, permitting positive feelings and a sense of wholeness^28^. In the consulted literature, this relation had not been assessed in an emerging adult population with low-risk alcohol consumption conditions, making this the first study to address that aspect. 

In the second model developed for the sake of respecification after the goodness-of-fit, two latent variables were established: spiritual transcendence and psychosocial wellbeing, constructed through the articulation of the variables measured in the study and the theoretical reflection deriving from Reed's theoretical proposal; in addition, the relation between the variables "vulnerability to consumption" and "spiritual transcendence" was measured. The relation between vulnerability to alcohol consumption and spiritual transcendence was also inverse, that is, to a low vulnerability to consumption corresponds a greater spiritual transcendence in youth; theoretically, the support for this relation follows the same sense as in the first model. While the relation between spiritual transcendence and psychosocial well-being was positive and statistically significant, this relation coincides with what Reed^7^ presents in her theory^1^, as well as with findings from other studies^15^ ^,^ ^29^. 

From the theoretical viewpoint, the latent variable "spiritual transcendence" was constructed based on the theoretical premises of self-transcendence and the spirituality model. The theory of self-transcendence was developed based on the acknowledgement of human beings' development and the philosophical belief in each person's potential to achieve well-being. That is how spiritual transcendence is defined in this study as "the natural and developmental capacity of people the extend the internal and relational limits in a spiritual context that allows them to achieve harmony, piece and wellbeing". The attributes of the variable are: self-transcendence, spiritual needs, spiritual practices, self-awareness and spiritual beliefs. 

The latent variable "psychosocial wellbeing" was derived from the proposals by Ryff^30^, considering psychological wellbeing as a progression of the continuous growth in the course of life, which involves working to comply with the purposes planned for one's existence, self-accomplishment, individualization and maturity, and which centers on mental health and positive functioning^30^. And "social wellbeing" was considered to be "the circumstances and functioning in society" of what Keyes proposed^17^. As a matter of fact, the following definition was proposed for this latent variable: "Psychosocial wellbeing is the expression of growth, maturity and the harmonious performance of the person in society, it is the demonstration of the individual strength of human beings and the potentials of positive social functioning"; its attributes are: Autonomy, Positive relationships with others, Purpose in life, Self-acceptance, Mastery of the environment, Personal growth, Social integration, Social acceptance, Social contribution, Social actualization and Social coherence. 

Based on the theoretical proposal of Self-transcendence, next, the relations with the phenomenon of non-consumption and moderate alcohol consumption should be discussed. Although Reed's Theory of Self-transcendence^1^ does not explicitly explain the mechanisms immersed in moderate alcohol consumption and non-consumption, the mechanisms can be outlined which people adopt in the development process that takes place in the course of life to achieve wellbeing and health. Hence, the study phenomenon is linked to the theory when it is evidenced that these two conducts, moderate consumption and non-consumption, are part of the expression of well-being and health, as has been described from such innovative perspectives as genospirituality^31^. From the perspective of the consumption patterns included in this study, these demonstrate lower risks and damage for people^32^. Similarly, well-being is conceived as a feeling of wholeness and health, it means being complete and being oneself. Thus, the emerging adults evidenced, through the coefficients of the variables measured, what they are, believe and think about their social and psychological wholeness in a context of hazardous alcohol consumption like the college world. 

The variable "vulnerability to alcohol consumption" evidenced the events the level of alcohol consumption brought about in the emerging adults. As signaled, in this particular group, low coefficients were found. Therefore, in line with the theory, these young people did not let the circumstances in life that led them towards alcohol consumption discourage them. On the opposite, they achieved internal development towards a renewed sense of identity and expansion of their personal limits, as demonstrated in the level of self-transcendence registered.

## Conclusions 

The relations among the variables vulnerability, self-transcendence and well-being proposed in Reed's theory are supported by the present findings. An expanded model is presented with two latent variables: spiritual well-being and psychosocial well-being; spiritual transcendence explains 23.2% of the variance in psychosocial well-being. An inverse relationship was found between vulnerability to consumption and spiritual self-transcendence, which demonstrates the protective role of human beings' internal development to maintain conducts that contribute to states of well-being and health. The psychosocial well-being present in the young adult population with low-risk consumption to a certain extent results from the expansion of personal, relational and time limits they have reached by counting on a clear sense in life and by finding meaning in the conducts considered part of their spirituality.

## Contributions to the discipline 

For the nurses, from the viewpoint of the discipline, it is very important to empirically test the theories that explain the phenomena they are interested in using structural equations. This statistical resource supports the nurses' theoretical reflections on their professional know-how with evidences. This study particularly reveals new routes to take care of young adults, with a view to supporting interventions and projects aimed at strengthening the spiritual sphere and self-transcendence in this population group. 

Health promotion programs and interventions departing from the perspective proposed should motivate the nurses to focus on emerging adults as people with possibilities, potentials (such as spiritual transcendence) and valid experiences, meanings constructed in daily life, which are the most important input to achieve higher levels of wellbeing. Based on these same young people, interventions should be proposed in a concerted instead of an enforced manner. 

 Studies are recommended that prove the effect of activities that support enhanced self-transcendence and its effect on health promotion in this population group. To confirm the relationships evidenced in this study, longitudinal studies are needed in the future, which involve groups of emerging adults from other universities and other contexts, such as workers. It should be investigated whether the relation between vulnerability to consumption in binge drinkers changes or continues in the same sense; in addition, it should be confirmed whether the differences and the relations between all variables are similar, comparing non-consuming emerging adults with binge drinkers of the same age. The literature mentions distinctive aspects among the variables included in the study according to gender. These analyses go beyond the focus of this study, which was the level of alcohol consumption instead of gender, another aspect for future investigation. 

## Study limitations 

The cross-sectional design can be considered a limitation, as this type of study reduces the strength of the causal relations among the variables studied. The type of sample at a single university and in a single city makes it not that easy to generalize the results. In addition, as the study was developed in a college context, the data may not be extendable to all emerging adults.
